# Structure of the human telomere in Na^+^ solution: an antiparallel (2+2) G-quadruplex scaffold reveals additional diversity

**DOI:** 10.1093/nar/gkt771

**Published:** 2013-08-31

**Authors:** Kah Wai Lim, Veronica Chinn Min Ng, Nerea Martín-Pintado, Brahim Heddi, Anh Tuân Phan

**Affiliations:** ^1^School of Physical and Mathematical Sciences, Nanyang Technological University, 637371 Singapore, ^2^School of Biological Sciences, Nanyang Technological University, 637551 Singapore and ^3^Instituto de Química Física Rocasolano, CSIC, 28006 Madrid, Spain

## Abstract

Single-stranded DNA overhangs at the ends of human telomeric repeats are capable of adopting four-stranded G-quadruplex structures, which could serve as potential anticancer targets. Out of the five reported intramolecular human telomeric G-quadruplex structures, four were formed in the presence of K^+^ ions and only one in the presence of Na^+^ ions, leading often to a perception that this structural polymorphism occurs exclusively in the presence of K^+^ but not Na^+^. Here we present the structure of a new antiparallel (2+2) G-quadruplex formed by a derivative of a 27-nt human telomeric sequence in Na^+^ solution, which comprises a novel core arrangement distinct from the known topologies. This structure complements the previously elucidated basket-type human telomeric G-quadruplex to serve as reference structures in Na^+^-containing environment. These structures, together with the coexistence of other conformations in Na^+^ solution as observed by nuclear magnetic resonance spectroscopy, establish the polymorphic nature of human telomeric repeats beyond the influence of K^+^ ions.

## INTRODUCTION

Telomeres, the ends of linear eukaryotic chromosomes, perform vital roles in the maintenance of genome integrity and the regulation of cell proliferation (1). Human telomeric DNA consists of thousands of tandem repeats of (TTAGGG)_n_ sequences (2,3), terminating as single-stranded DNA of 100–200 nt at the 3′-ends (4). Such guanine-rich repeats are capable of adopting four-stranded G-quadruplex structures (5–8) under physiological conditions. G-quadruplex formation at the telomeric ends has been shown to inhibit the activity of the enzyme telomerase (9), which is essential for the proliferation of most cancer cells (10,11). Insights from high-resolution G-quadruplex structures adopted by these sequences could thus aid in drug design efforts targeting telomeric DNA for potential anticancer treatment (12,13).

To date, at least five distinct intramolecular G-quadruplex folding topologies (Supplementary Figure S1) have been reported for natural human telomeric repeats (14–23), four of which were observed in the presence of K^+^ ions. Form 1 (15–17) and Form 2 (18,19), observed in K^+^ solution, both comprise the (3+1) G-tetrad core, but differ in procession of the different loops; Form 3 (20), also observed in K^+^ solution, consists of a two-G-tetrad basket-type core with extensive base stacking interactions in the loops. In a K^+^-containing crystal (21) and under water-depleted conditions (22,24–26), the all-parallel-stranded propeller-type G-quadruplex structure was shown to be the preferred form. On the other hand, the only conformation to have been characterized in Na^+^ solution consists of a three-layered basket-type G-quadruplex (23). Myriad studies on human telomeric G-quadruplexes have been carried out using a wide variety of techniques (27–41), ranging from ultraviolet (UV) (27), circular dichroism (CD) (28), fluorescence (29–31), Raman (32), electron paramagnetic resonance (33) and nuclear magnetic resonance (NMR) spectroscopy (34), to X-ray crystallography (21), mass spectrometry (35), gel electrophoresis (36), single-molecule Förster resonance energy transfer (37,38), laser-tweezers pulling (39), calorimetry (40) and molecular dynamics (MD) simulations (41), and the results have revealed the polymorphic nature of human telomeric repeats in the presence of K^+^ ions. While not fully addressed, this structural polymorphism has also been implicated in Na^+^ solution (29,36,37), yet the basket form has largely been used as the sole reference structure for interpretation of studies under Na^+^-containing environment. Knowledge on the structural disparities, if any, in the presence of Na^+^ ions would contribute toward fundamental understanding of the cationic influence on human telomeric repeats under different contexts. Using NMR, here we show that four-repeat human telomeric sequences could adopt multiple G-quadruplex conformations in Na^+^ solution. We present the solution structure of one of these forms, which exhibits features distinct from the known topologies. This latest addition to the ensemble of telomeric G-quadruplex structures establishes the polymorphic nature of human telomeric repeats beyond the influence of K^+^ ions, and builds on the available pool of structural motifs for targeting of these important entities.

## METHODS

### Sample preparation

Unlabeled (Supplementary Table S1) and site-specific labeled (2%-^15^N-enriched or ^2^H-labeled; Supplementary Table S2) DNA oligonucleotides were chemically synthesized on an ABI 394 DNA/RNA synthesizer using products from Glen Research, Spectra Gases and Cambridge Isotope Laboratories. The oligonucleotides were de-protected following the manufacturer’s protocols and purified using Poly-Pak™ cartridges. Samples were dialyzed successively against water, ∼25 mM NaCl solution and water again. They were subsequently frozen, lyophilized and suspended in a buffer containing 20 mM sodium phosphate (pH 7.0) and 70 mM NaCl. DNA concentration is expressed in strand molarity using a nearest-neighbor approximation for the absorption coefficients of the unfolded species (42). The same extinction coefficient was used for the natural and ^Br^G-substituted oligonucleotides.

### UV spectroscopy

Thermal stability of different DNA oligonucleotides was characterized by recording the UV absorbance at 295 nm (43) as a function of temperature (20–90°C) using a JASCO V-650 spectrophotometer. The heating and cooling rates were 0.2°C/min. Two baselines corresponding to the completely folded (low temperature) and completely unfolded (high temperature) states were manually drawn to determine the fractions of folded and unfolded species during the melting/folding transition. The melting temperature (*T*_m_) is defined as the temperature for which there are equal fractions of folded and unfolded species. Experiments were performed with quartz cuvettes (1-cm pathlength for low DNA concentrations and 0.2-cm pathlength for high DNA concentrations).

### CD spectroscopy

CD spectra were recorded at 20°C on a JASCO-815 spectropolarimeter over the range of 220–320 nm using a 1-cm pathlength quartz cuvette with a reaction volume of 600 µl. For each sample, an average of three scans was taken, the spectrum of the buffer was subtracted and the data were zero-corrected at 320 nm.

### NMR spectroscopy

Strand concentration of the NMR samples was typically 0.5–2.0 mM. NMR experiments were performed on Bruker AVANCE 600- and 700-MHz spectrometers at 25°C, unless otherwise specified. Resonances for guanine residues were assigned unambiguously using site-specific low-enrichment ^15^N labeling (44), site-specific ^2^H labeling (45) and through-bond correlations at natural abundance (46,47). Spectral assignments were assisted by NOESY, COSY, TOCSY and {^1^H-^13^C}-HSQC experiments. The spectra were processed with the software TopSpin and analyzed using the program FELIX (Felix NMR, Inc.).

### Structure calculation

Inter-proton distances for the d[(TTAGGG)_3_TTA(^Br^G)GGTTA] (*htel27[Br22]*) G-quadruplex were deduced from NOESY experiments performed in H_2_O (mixing time, 200 ms) and D_2_O (mixing times, 100 and 350 ms). Structures were calculated based on distance-restrained molecular dynamics refinement following distance geometry simulated annealing using the program XPLOR-NIH (48). Hydrogen bond restraints, inter-proton distance restraints, dihedral restraints, planarity restraints and repulsive restraints were imposed during structure calculations. Structures were displayed using the program PyMOL (49). Detailed procedures for structure calculation are described in Supplementary Text.

### Data deposition

The coordinates for the d[(TTAGGG)_3_TTA(^Br^G)GGTTA] (*htel27[Br22]*) G-quadruplex have been deposited in the Protein Data Bank (accession code 2MBJ).

## RESULTS AND DISCUSSION

### Four-repeat human telomeric sequences adopt diverse G-quadruplex conformations in Na^+^ solution

**Figure 1.**
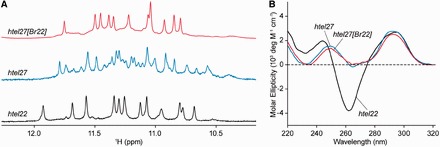
(**A**) NMR imino proton spectra and (**B**) CD spectra of human telomeric sequences: *htel22*, *htel27* and *htel27[Br22]* in ∼100 mM Na^+^.

NMR spectra of four-repeat natural human telomeric sequences with different flanking ends (Supplementary Table S1) in Na^+^ solution indicated the adoption of multiple conformations (Supplementary Figure S2). In agreement with the previous study (23), the sequence d[AGGG(TTAGGG)_3_] (denoted as *htel22*; Table 1) displayed 12 major imino proton peaks at ∼10.6–12.0 ppm (Figure 1A), corresponding to the establishment of the basket-type G-quadruplex (Supplementary Figure S1A). In addition, two sequences with the same 5′-flanking end as *htel22* showed a major species with a highly similar spread of imino proton peaks (Supplementary Figure S2F and G), suggesting their adoption of the same topology as the prevailing form. The other sequences mostly displayed the coexistence of two or more major species, judging from the number and intensity of imino proton peaks. CD spectra of all 16 sequences displayed two positive maxima at ∼250 and ∼295 nm, and a trough at ∼260–270 nm (Supplementary Figure S3). The 295-nm peak is characteristic of opposite-polarity stacking of G-tetrads (50), suggesting that these sequences largely conform to antiparallel G-quadruplexes in Na^+^ solution. Note that *htel22* produced a sharp negative trough at ∼260 nm (Figure 1B), which was less intense in the other sequences.

**Table 1.** Representative natural and modified human telomeric sequences used in this study^a^

Name	Sequence
*htel22*	A GGG TTA GGG TTA GGG TTA GGG
*htel27*	TTA GGG TTA GGG TTA GGG TTA GGG TTA
*htel27[Br22]*	TTA GGG TTA GGG TTA GGG TTA **^Br^****G**GG TTA

^a^The modified 8-bromoguanine (^Br^G) residue is in boldface.

### Formation of a G-quadruplex with an alternative conformation distinct from *htel22*

We chose to probe the structures present in the sequence d[(TTAGGG)_4_TTA] (denoted as *htel27*; Table 1) (Figure 1A), which has the largest number of flanking nucleotides for potential interactions. Previously, 8-bromoguanine (^Br^G)-for-G substitution has been used to drive the folding of an oligonucleotide toward a single major G-quadruplex structure by favoring the *syn* glycosidic conformation at the substituted residue (17,18,20,51). Here, single ^Br^G incorporation at position G22 of *htel27*, giving rise to the sequence d[(TTAGGG)_3_TTA(^Br^G)GGTTA] (denoted as *htel27[Br22]*; Table 1), led to the emergence of a predominant species (>85%) amenable for detailed structural characterization (Figure 1A). *Htel27[Br22]* showed 12 major imino proton peaks at ∼10.8–11.8 ppm, indicating the adoption of a three-layered G-quadruplex. The CD spectra of *htel27[Br22]* and *htel27* were highly similar (Figure 1B), suggesting that *htel27[Br22]* could correspond to the preexisting major conformation(s) in *htel27*, or that the core topologies of *htel27[Br22]* and *htel27* were closely related. Their CD spectra were substantially different from that of *htel22*, notably the absence of the sharp negative trough at ∼260 nm, pointing to the adoption of an alternative folding topology. Melting analyses of the three sequences were carried out by monitoring the UV absorbance at 295 nm (43), and the melting temperatures (*T*_m_) of *htel22*, *htel27* and *htel27[Br22]* were found to be 57.6, 44.5 and 50.8°C, respectively, in ∼100 mM Na^+^ (Supplementary Figure S4). The ^Br^G-substituted oligonucleotide displayed a *T*_m_ ∼6°C higher than that of the natural counterpart, consistent with observations from previous studies (20,51).

### NMR spectral assignments

**Figure 2.**
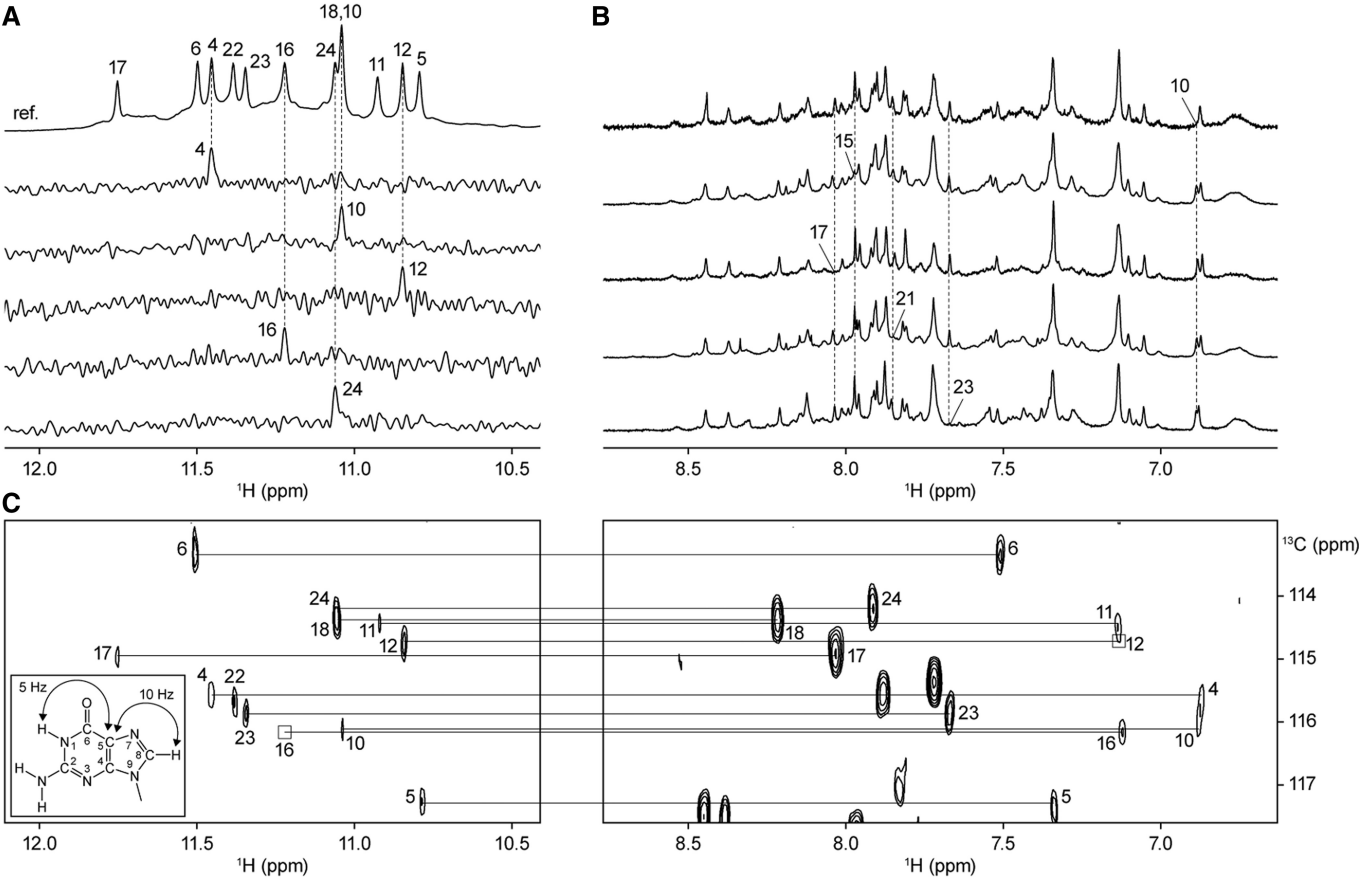
NMR spectral assignments of *htel27[Br22]* in Na^+^ solution. (**A**) Guanine imino protons were assigned based on^15^N-filtered spectra of samples, 2% ^15^N-labeled at the indicated positions. The reference spectrum (ref.) with the respective guanine imino proton assignments is shown at the top. (**B**) Examples of adenine and guanine H8 proton assignments through site-specific ^2^H labeling at the indicated positions. (**C**) Through-bond correlations between guanine imino and H8 protons via ^13^C5 at natural abundance, using long-range *J*-couplings shown in the inset. Missing/weak correlations are framed in boxes.

To proceed with the structural elucidation of *htel27[Br22]*, guanine imino (Figure 2A) and adenine/guanine H8 (Figure 2B) protons were unambiguously assigned using site-specific low-enrichment ^15^N labeling (44) and site-specific ^2^H labeling (45), respectively (Supplementary Table S2). The guanine imino and H8 proton assignments were further corroborated by through-bond correlations at natural abundance (46) (Figure 2C). Assignments of thymine residues were supplemented by T-to-U substitutions (Supplementary Table S2). The H8/H6-H1′ NOE sequential connectivity of *htel27[Br22]* (Supplementary Figure S5) was completed with the assistance of other through-bond correlation experiments (COSY, TOCSY and {^1^H-^13^C}-HSQC; data not shown) (47). The strong intensity of intraresidue H8-H1′ NOE cross-peaks for G4, G5, G10, G16 and G23 (Supplementary Figure S6) indicated that these guanine residues, together with ^Br^G22, adopt the *syn* glycosidic conformation. All other guanine residues adopt the *anti* glycosidic conformation.

### *Htel27[Br22]* adopts a new antiparallel (2+2) folding topology in Na^+^ solution

**Figure 3.**
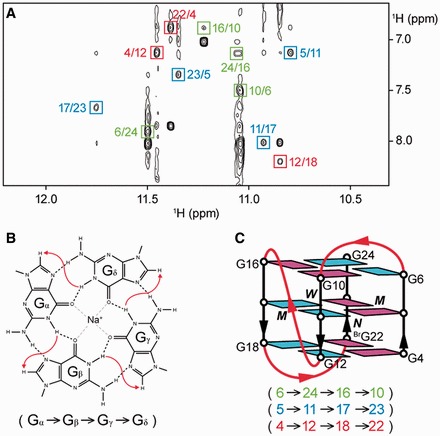
Determination of the folding topology of *htel27[Br22]*. (**A**) NOESY spectrum (mixing time, 200 ms) showing the imino–H8 connectivity of *htel27[Br22]*. Cross-peaks that identify the arrangement of the G-tetrads are framed and labeled with the residue number of imino protons in the first position and that of H8 protons in the second position. (**B**) Characteristic guanine imino–H8 NOE connectivity patterns around a G_α_•G_β_•G_γ_•G_δ_ tetrad as indicated with arrows (connectivity between G_δ_ and G_α_ implied). (**C**) Schematic structure of *htel27[Br22]*. *Anti* guanines are colored in cyan, while *syn* guanines and 8-bromoguanine are colored in magenta. The backbones of the core and loops are colored in black and red, respectively. *W*, *M* and *N* represent wide, medium and narrow grooves, respectively.

Having determined the guanine imino and H8 proton assignments for *htel27[Br22]*, the alignment of the tetrads could then be deduced based on characteristic cyclic imino–H8 NOE connectivity patterns within each tetrad (Figure 3A and B). The top G-tetrad (G6•G24•G16•G10) is oriented in the opposite hydrogen-bond directionality with respect to the other two G-tetrads, (G5•G11•G17•G23) and (G4•G12•G18•^Br^G22), and the glycosidic conformations of guanines are *anti•anti•syn•syn* for all three tetrads. The central positioning of (G5•G11•G17•G23) in the tetrad core agrees with solvent exchange data showing that the imino protons from this G-tetrad were the most protected (Supplementary Figure S7). Connecting the sequential guanine residues and joining the corners of the G-tetrad core with the linking TTA loops, we derived an antiparallel G-quadruplex scaffold consisting of two adjacent strands pointing up and two remaining strands pointing down (up–up–down–down) or a (2+2) G-quadruplex scaffold (14) with a successive loop arrangement edgewise–propeller–edgewise (Figure 3C).

### Na^+^ solution structure of the *htel27[Br22]* G-quadruplex

**Figure 4.**
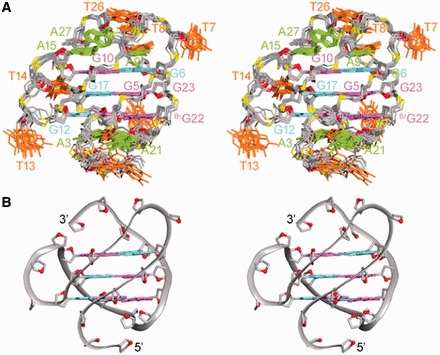
Stereoviews of the *htel27[Br22]* G-quadruplex structure in Na^+^ solution. (**A**) Ten superimposed refined structures. (**B**) Ribbon view of a representative structure. *Anti* guanines are colored in cyan; *syn* guanines and 8-bromoguanine, magenta; adenines, green; thymines, orange; backbone and sugars, gray; O4′ atoms, red; phosphorus atoms, yellow.

The structure of *htel27[Br22]* G-quadruplex in Na^+^ solution (Figure 4) was computed on the basis of NMR restraints (Table 2). There are one wide, one narrow and two medium grooves. The first (edgewise) loop crosses the top of the narrow groove, the second (propeller) loop traverses across one of the medium grooves, while the third (edgewise) loop completes a turn over the bottom of the wide groove. The 5′- and 3′-flanking TTA nucleotides take part in hydrogen-bond and stacking interactions at the bottom and the top of the structure, respectively. There is a Watson–Crick A9•T25 base pair capping the top of the tetrad core. A15 from the propeller loop also stacks over the top G-tetrad, potentially establishing interactions with the loop elements at the top. T14 from the same loop projects into the medium groove, whereas T13 flips out of the propeller loop. At the bottom of the structure, A3 and T20 establishes a Watson–Crick base pair, with A21 completing the formation of a triple platform. These stabilizing interactions of the loop and terminal residues would agree with the observation of additional broad imino proton peaks at ∼12.2–14.0 ppm (Supplementary Figure S8). The establishment of base pair stacking interactions across the ends of the tetrad core could have provided considerable contribution toward the adoption of the major conformation of *htel27[Br22]*, consistent with previous observations regarding other human telomeric G-quadruplex structures formed in K^+^ solution (15–20). In a longer sequence context, the various structural forms could exist in a state of equilibrium, with possible interconversion among them.

**Table 2.** Statistics of the computed structures of the 27-nt ^Br^G-modified human telomeric sequence d[(TTAGGG)_3_TTA(^Br^G)GGTTA] (*htel27[Br22]*)^a^

A. NMR restraints		
Distance restraints	D_2_O	H_2_O
Intraresidue distance restraints	446	0
Sequential (*i*, *i* + 1) distance restraints	197	26
Long-range (*i*, ≥ *i* + 2) distance restraints	79	32
Other restraints		
Hydrogen bond restraints	48	
Dihedral restraints	12	
Planarity restraints	3	
Repulsive restraints^b^	2	
B. Structure statistics for 10 molecules following distance-restrained MD refinement		
NOE violations		
Number (>0.2 Å)	0.800 ± 0.872	
Maximum violation (Å)	0.195 ± 0.050	
RMSD of violations (Å)	0.022 ± 0.002	
Deviations from the ideal covalent geometry		
Bond lengths (Å)	0.003 ± 0.000	
Bond angles (°)	0.694 ± 0.007	
Impropers (°)	0.367 ± 0.004	
Pairwise all heavy atom RMSD values (Å)		
All heavy atoms except T1, T2	1.04 ± 0.12	
All heavy atoms	1.49 ± 0.35	

^a^PDB ID: 2MBJ.

^b^Distance restraints between pairs of protons that do not exhibit NOE cross-peaks.

### Novel features of the *htel27[Br22]* G-quadruplex

We have shown that in Na^+^ solution, *htel27[Br22]* assumes a novel antiparallel (2+2) folding topology (Figure 3C). Although the tetrad cores of *htel27[Br22]* (this work) and *htel22* (23) possess the same relative strand orientations, they differ in the hydrogen-bond directionalities of the tetrads: anticlockwise–clockwise–clockwise for *htel27[Br22]* versus clockwise–anticlockwise–clockwise for *htel22* (Supplementary Figure S9). Loop arrangement of *htel27[Br22]* (edgewise–propeller–edgewise) is also different from that of *htel22* (edgewise–diagonal–edgewise). To date, antiparallel (2+2) G-quadruplexes that have been investigated largely consist of edgewise and/or diagonal loops (20,23,52–54), while quadruplexes containing propeller loops mostly belong to the all-parallel-stranded (21,22) or (3+1) (15–19,55–57) core topology. Antiparallel (2+2) G-quadruplexes with a propeller loop (58), such as that formed by *htel27[Br22]*, have not been observed in the context of human telomeric repeats.

## CONCLUSION

The antiparallel (2+2) G-quadruplex scaffold adopted by *htel27[Br22]* (and presumably a subpopulation of *htel27*), as well as the observation of the coexistence of multiple conformations in the series of four-repeat natural human telomeric sequences in Na^+^ solution, point to additional diversity than had been previously assumed. Even though intracellular Na^+^ content is ∼10 times lower than that of K^+^, these structures observed in Na^+^ solution could potentially take part in the interconversion between various quadruplex forms.

## ACCESSION NUMBER

PDB: accession code 2MBJ.

## SUPPLEMENTARY DATA

Supplementary Data are available at NAR Online.

## FUNDING

Singapore Ministry of Education and Nanyang Technological University (to A.T.P.). Funding for open access charge: Singapore Ministry of Education.

*Conflict of interest statement*. None declared.

## Supplementary Material

Supplementary Data
