# Supplementary material for: Structure of the human telomere in Na+ solution: an antiparallel (2+2) G-quadruplex scaffold reveals additional diversity
Source: Nucleic Acids Res. 2013 Aug 31;41(22):10556–62. doi: 10.1093/nar/gkt771 (PMC3905899; doi:10.1093/nar/gkt771)
Supplement: Supplementary Data [file supp_41_22_10556__index.html]

Structure of the human telomere in Na+ solution: an antiparallel (2+2) G-quadruplex scaffold reveals additional diversity — Structure of the human telomere in Na+ solution: an antiparallel (2+2) G-quadruplex scaffold reveals additional diversity — Supplementary Data 

# Structure of the human telomere in Na+ solution: an antiparallel (2+2) G-quadruplex scaffold reveals additional diversity

## Supplementary Data

files

**Files in this Data Supplement:**

- Supplementary Data - pdf file
