# Supplementary material for: Structure of the human telomere in Na+ solution: an antiparallel (2+2) G-quadruplex scaffold reveals additional diversity
Source: Nucleic Acids Res. 2013 Aug 31;41(22):10556–62. doi: 10.1093/nar/gkt771 (PMC3905899; doi:10.1093/nar/gkt771)
Supplement: Supplementary Data [file supp_gkt771_nar-01523-f-2013-File002.pdf]

Structure of the human telomere in Na^+ solution: an antiparallel (2+2) G-quadruplex scaffold reveals additional diversity

Kah Wai Lim[1,2], Veronica Chinn Min Ng[1], Nerea Martín-Pintado[1,3], Brahim Heddi[1] and Anh Tuân Phan[1,*]

[1]School of Physical and Mathematical Sciences,
Nanyang Technological University, Singapore

[2]School of Biological Sciences,
Nanyang Technological University, Singapore

[3]Instituto de Química Física "Rocasolano", CSIC, Madrid, Spain

SUPPLEMENTARY DATA

SUPPLEMENTARY TEXT

Methods for structure calculation

Hydrogen-Bond Restraints. Each hydrogen bond was restrained by two distances (donor atom to acceptor atom and proton to acceptor atom) which correspond to ideal hydrogen bond geometry. The force constant for hydrogen bond restraints was kept at 32 $kcal.mol^{-1}.Å^{-2}$ throughout the computation.

Non-Exchangeable Proton Distance Restraints. Distances between non-exchangeable protons were deduced from NOESY spectra in D_2O (mixing times, 100 and 350 ms) and implemented as distance restraints during structure calculations. These interproton distance restraints were manually classified as very strong (1.80–3.00 Å), strong (2.25–3.75 Å), medium (2.85–4.75 Å), weak (3.60–6.00 Å), or very weak (4.50–7.50 Å). Heavily overlapped cross-peaks were given more permissible bounds of 1.80–4.60 Å or 3.50–7.50 Å. Cross-peaks involving a methyl group were interpreted as distances to the methyl carbon. The upper bounds for these peaks were increased by 0.5 Å to account for the radius of the methyl group. The upper bounds for cross-peaks involving ambiguous atom selection were also increased by 0.5 Å.

Exchangeable Proton Distance Restraints. Cross-peaks involving exchangeable protons were obtained from NOESY spectrum in H_2O at 200-ms mixing time. The peaks were manually classified as very strong (1.8–3.2 Å), strong (2.4–4.0 Å), medium (3.0–5.4 Å), weak (3.6–6.4 Å), or very weak (4.2–7.8 Å). Heavily overlapped cross-peaks were given more permissible bounds of 1.80–4.50 Å or 3.80–7.80 Å. Cross-peaks involving a methyl group were interpreted as distances to the methyl carbon. The upper bounds for these peaks were increased by 0.5 Å to account for the radius of the methyl group. The upper bounds for cross-peaks involving ambiguous atom selection were also increased by 0.5 Å.

Dihedral Restraints. The glycosidic χ torsion angle for experimentally-determined *syn* guanine residues were restrained to 60°, while that for *anti* guanine residues were fixed at 240°. The bounds for these dihedrals were set at ±40° (central tetrad) or ±70° (outer tetrads).

Planarity Restraints. Planarity restraints were enforced on the base atoms for the tetrads.

Repulsive Restraints. Repulsive restraints (4.2–80 Å) were applied on pairs of protons that do not exhibit cross-peaks in NOESY.

Distance Geometry Simulated Annealing. An extended conformation of the sequence d[(TTAGGG)$_3$-TTA(^{Br}G)GGTTA] with ideal geometry was generated randomly using XPLOR-NIH (48). The extended DNA was then subjected to distance geometry simulated annealing (DGSA) by supplying the full set of hydrogen-bond, distance, dihedral, planarity and repulsive restraints. 100 structures were generated and subjected to further refinements.

Distance-Restrained Molecular Dynamics Refinement. All 100 DGSA structures were refined with distance-restrained molecular dynamics. The system was heated from 300 K to 1000 K in 14 ps and allowed to equilibrate for 6 ps, wherein the force constants for the distance restraints were kept at 2 kcal.mol^{-1}.Å^{-2}. The force constants for non-exchangeable proton, exchangeable proton, and repulsive distance restraints were then scaled to final values of 16, 8, and 8 kcal.mol^{-1}.Å^{-2}, respectively, in a 20-ps interval and allowed to equilibrate for a further 200 ps. Subsequently, the system was slowly cooled down to 300 K in 42 ps, after which equilibration was performed for another 18 ps. Coordinates of the molecule were saved every 0.5 ps during the last 4 ps and averaged. In the final step, the average structure was subjected to minimization until the gradient of energy was less than 0.1 kcal.mol-1. Dihedral (50 kcal.mol^{-1}.rad^{-2}) and planarity (2 kcal.mol^{-1}.Å^{-2} for the tetrads) restraints were maintained throughout the course of refinement. 10 best structures with the lowest overall energy were selected. Structures were displayed using the program PyMOL (49).

SUPPLEMENTARY TABLES

Table S1. Natural and modified human telomeric sequences used in this study

	Name	Sequence[a]
Natural	*4TTA01*	GGG TTA GGG TTA GGG TTA GGG
	4TTA02	GGG TTA GGG TTA GGG TTA GGG T
	4TTA03	GGG TTA GGG TTA GGG TTA GGG TT
	4TTA04	GGG TTA GGG TTA GGG TTA GGG TTA
	4TTA05/htel22	A GGG TTA GGG TTA GGG TTA GGG
	4TTA06	A GGG TTA GGG TTA GGG TTA GGG T
	4TTA07	A GGG TTA GGG TTA GGG TTA GGG TT
	4TTA08	A GGG TTA GGG TTA GGG TTA GGG TTA
	4TTA09	TA GGG TTA GGG TTA GGG TTA GGG
	4TTA10	TA GGG TTA GGG TTA GGG TTA GGG T
	4TTA11	TA GGG TTA GGG TTA GGG TTA GGG TT
	4TTA12	TA GGG TTA GGG TTA GGG TTA GGG TTA
	4TTA13	TTA GGG TTA GGG TTA GGG TTA GGG
	4TTA14	TTA GGG TTA GGG TTA GGG TTA GGG T
	4TTA15	TTA GGG TTA GGG TTA GGG TTA GGG TT
	4TTA16/htel27	TTA GGG TTA GGG TTA GGG TTA GGG TTA
^{Br}G-Modified	*4TTA16[Br4G]*	TTA (**^{Br}G**) GG TTA GGG TTA GGG TTA GGG TTA
	4TTA16[Br5G]	TTA G (**^{Br}G**) G TTA GGG TTA GGG TTA GGG TTA
	4TTA16[Br10G]	TTA GGG TTA (**^{Br}G**) GG TTA GGG TTA GGG TTA
	4TTA16[Br11G]	TTA GGG TTA G (**^{Br}G**) G TTA GGG TTA GGG TTA
	4TTA16[Br16G]	TTA GGG TTA GGG TTA (**^{Br}G**) GG TTA GGG TTA
	4TTA16[Br17G]	TTA GGG TTA GGG TTA G (**^{Br}G**) G TTA GGG TTA
	4TTA16[Br22G]/htel27[Br22]	TTA GGG TTA GGG TTA GGG TTA (**^{Br}G**) GGTTA
	4TTA16[Br23G]	TTA GGG TTA GGG TTA GGG TTA G (**^{Br}G**) GTTA
	4TTA16[Br5G][Br22G]	TTA G (**^{Br}G**) G TTA GGG TTA GGG TTA (**^{Br}G**) GGTTA

[a] The modified 8-bromoguanine (^{Br}G) residue is in boldface.

Table S2. Site-specific labeled DNA sequences used in this study

	Sequence[a,b,c]								
^{15}N-labeled	TTA	(*G)GG	TTA	GGG	TTA	GGG	TTA	(^{Br}**G**)GG	TTA
	TTA	GGG	TTA	(*G)GG	TTA	GGG	TTA	(^{Br}**G**)GG	TTA
	TTA	GGG	TTA	GG(*G)	TTA	GGG	TTA	(^{Br}**G**)GG	TTA
	TTA	GGG	TTA	GGG	TTA	(*G)GG	TTA	(^{Br}**G**)GG	TTA
	TTA	GGG	TTA	GGG	TTA	GGG	TTA	(^{Br}**G**)G(*G)	TTA
^{2}H-labeled	TT(#A)	GGG	TTA	GGG	TTA	GGG	TTA	(^{Br}**G**)GG	TTA
	TTA	(#G)GG	TTA	GGG	TTA	GGG	TTA	(^{Br}**G**)GG	TTA
	TTA	G(#G)G	TTA	GGG	TTA	GGG	TTA	(^{Br}**G**)GG	TTA
	TTA	GG(#G)	TTA	GGG	TTA	GGG	TTA	(^{Br}**G**)GG	TTA
	TTA	GGG	TT(#A)	GGG	TTA	GGG	TTA	(^{Br}**G**)GG	TTA
	TTA	GGG	TTA	(#G)GG	TTA	GGG	TTA	(^{Br}**G**)GG	TTA
	TTA	GGG	TTA	G(#G)G	TTA	GGG	TTA	(^{Br}**G**)GG	TTA
	TTA	GGG	TTA	GG(#G)	TTA	GGG	TTA	(^{Br}**G**)GG	TTA
	TTA	GGG	TTA	GGG	TT(#A)	GGG	TTA	(^{Br}**G**)GG	TTA
	TTA	GGG	TTA	GGG	TTA	(#G)GG	TTA	(^{Br}**G**)GG	TTA
	TTA	GGG	TTA	GGG	TTA	G(#G)G	TTA	(^{Br}**G**)GG	TTA
	TTA	GGG	TTA	GGG	TTA	GG(#G)	TTA	(^{Br}**G**)GG	TTA
	TTA	GGG	TTA	GGG	TTA	GGG	TT(#A)	(^{Br}**G**)GG	TTA
	TTA	GGG	TTA	GGG	TTA	GGG	TTA	(^{Br}**G**)(#G)G	TTA
	TTA	GGG	TTA	GGG	TTA	GGG	TTA	(^{Br}**G**)G(#G)	TTA
U-substituted	TTA	GGG	**U**TA	GGG	TTA	GGG	TTA	(^{Br}**G**)GG	TTA
	TTA	GGG	TTA	GGG	**U**TA	GGG	TTA	(^{Br}**G**)GG	TTA
	TTA	GGG	TTA	GGG	TTA	GGG	**U**TA	(^{Br}**G**)GG	TTA

[a]The modified 8-bromoguanine (^{Br}G) or uracil (U) residues are in boldface.
[b]Individual nucleotides 2%-^{15}N-labeled are marked by asterisks (*G).
[c]Individual nucleotides ^{2}H-labeled at the H8 position are marked by hash signs ($^{\#}$G).

SUPPLEMENTARY FIGURES

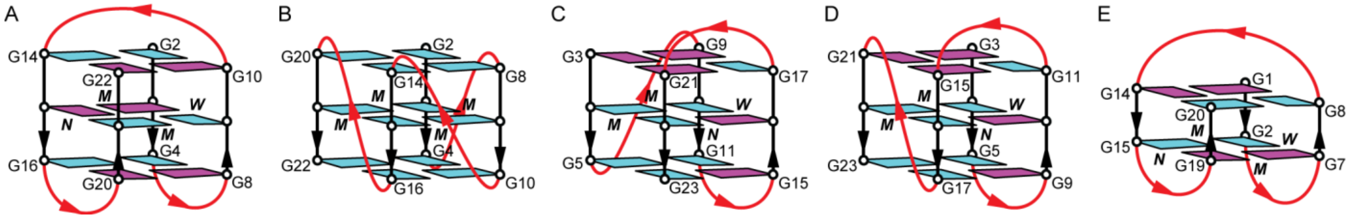

Figure S1. Schematic structures of various intramolecular G-quadruplex folding topologies adopted by human telomeric DNA sequences across different experimental conditions: (**A**) three-layered basket-type G-quadruplex adopted by d[AGGG(TTAGGG)$_3$] (*htel22*) in Na^+ solution (23), (**B**) propeller-type all-parallel-stranded G-quadruplex adopted by d[AGGG(TTAGGG)$_3$] (*htel22*) in K^+-containing crystal (21) and under molecular crowding-mimicking condition (22), (**C**) (3+1) G-quadruplex adopted by d[TAGGG(TTAGGG)$_3$] in K^+ solution (Form 1) (15-17), (**D**) (3+1) G-quadruplex formed by d[TAGGG(TTAGGG)$_3$TT] in K^+ solution (Form 2) (18,19), and (**E**) two-layered basket-type G-quadruplex formed by d[GGG(TTAGGG)$_3$T] in K^+ solution (Form 3) (20). *anti* and *syn* guanines are colored in cyan and magenta, respectively. The backbones of the core and loops are colored in black and red, respectively.

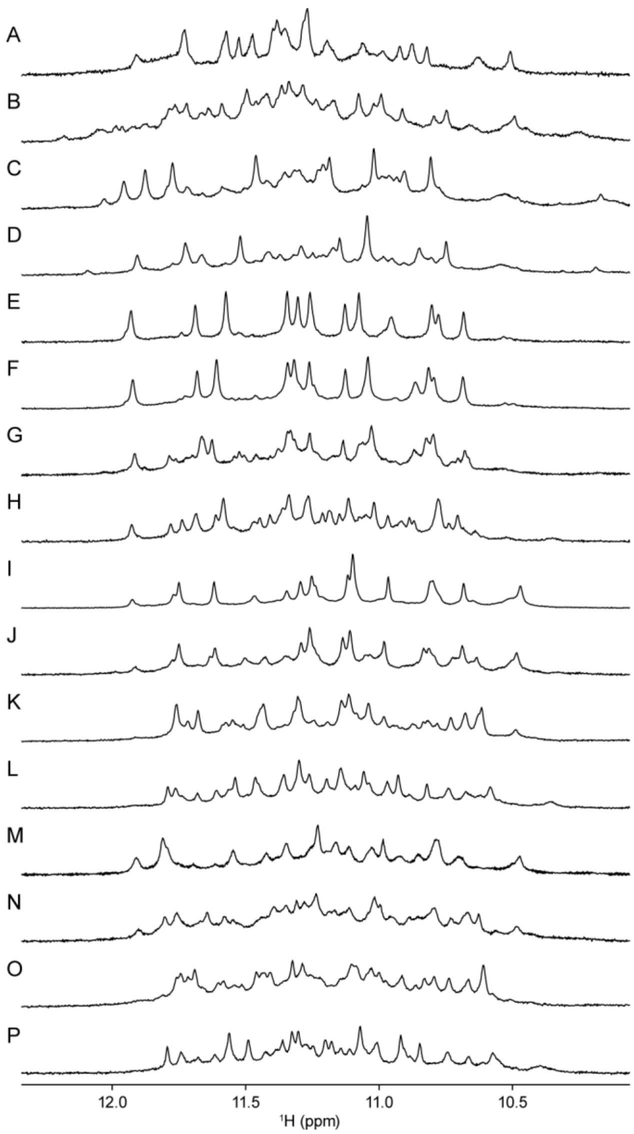

Figure S2. 1D NMR imino proton spectra of four-repeat natural human telomeric sequences in Na^+ solution: (**A**) *4TTA01*, (**B**) *4TTA02*, (**C**) *4TTA03*, (**D**) *4TTA04*, (**E**) *4TTA05*, (**F**) *4TTA06*, (**G**) *4TTA07*, (**H**) *4TTA08*, (**I**) *4TTA09*, (**J**) *4TTA10*, (**K**) *4TTA11*, (**L**) *4TTA12*, (**M**) *4TTA13*, (**N**) *4TTA14*, (**O**) *4TTA15*, and (**P**) *4TTA16*.

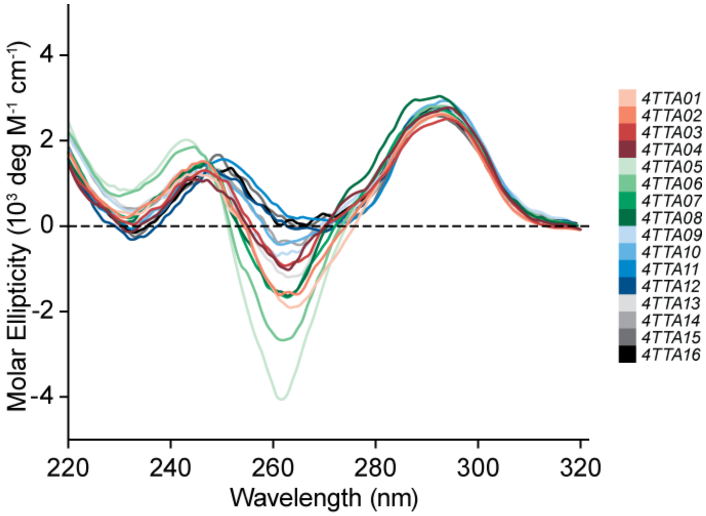

Figure S3. CD spectra of four-repeat natural human telomeric sequences in Na^{+} solution: (**A**) *4TTA01*, (**B**) *4TTA02*, (**C**) *4TTA03*, (**D**) *4TTA04*, (**E**) *4TTA05*, (**F**) *4TTA06*, (**G**) *4TTA07*, (**H**) *4TTA08*, (**I**) *4TTA09*, (**J**) *4TTA10*, (**K**) *4TTA11*, (**L**) *4TTA12*, (**M**) *4TTA13*, (**N**) *4TTA14*, (**O**) *4TTA15*, and (**P**) *4TTA16*. Color-coded on the right.

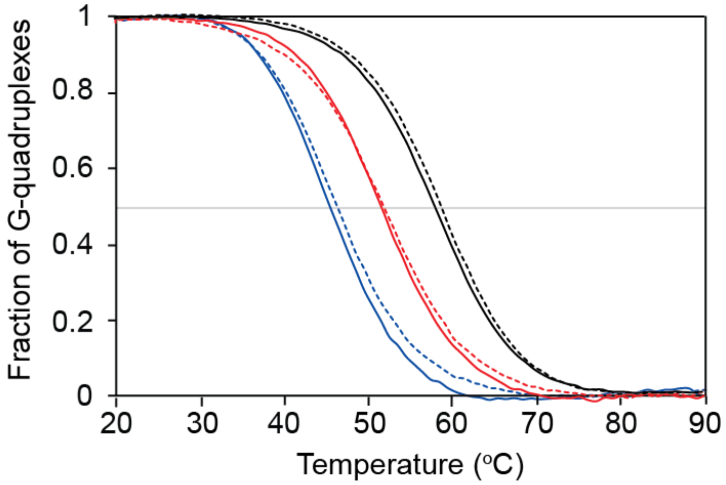

Figure S4. Fraction of G-quadruplexes as a function of temperature for *htel27* (blue), *htel27[Br22]* (red), and *htel22* (black), as monitored by UV absorbance at 295 nm (43). DNA concentration is 5 μM. Solid lines represent cooling curves while dotted lines represent heating curves.

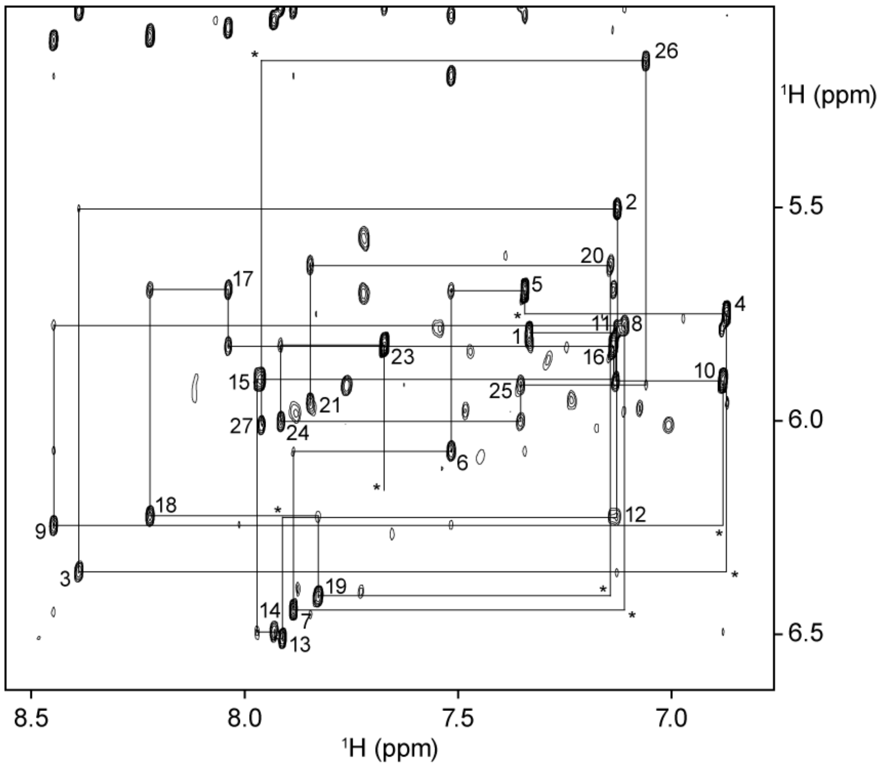

Figure S5. NOESY spectrum (mixing time, 350 ms) showing the H8/H6–H1’ NOE connectivity of *htel27[Br22]* in Na^+ solution. Intraresidue H8/H6–H1’ NOE cross-peaks are labeled with residue numbers. Weak or missing sequential connectivities are marked with asterisks. The connectivity is broken at position 22 because the H8 proton of G22 was replaced by bromine.

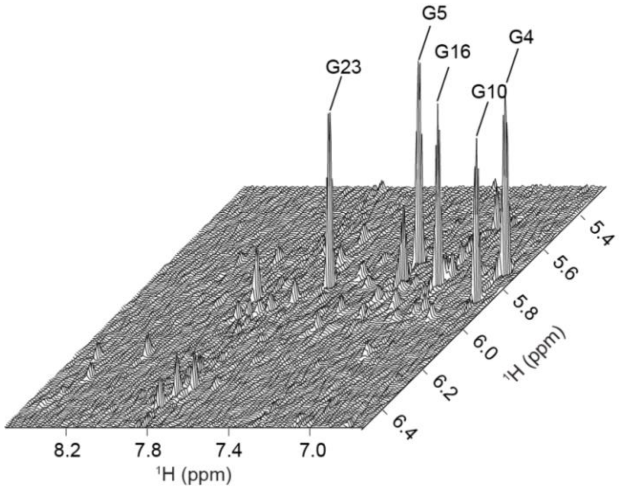

Figure S6. Stacked plot of the NOESY spectrum (mixing time, 350 ms) of *htel27[Br22]* in Na^+ solution. The five strong *syn* intraresidue H8–H1’ cross-peaks for G4, G5, G10, G16, and G23 are labeled.

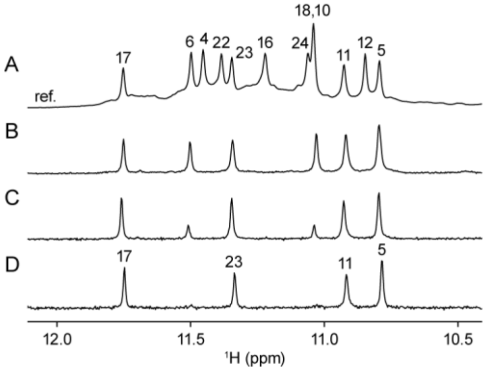

Figure S7. Solvent exchange of imino protons of *htel27[Br22]*. (**A**) The reference spectrum (ref.) in H_2O. (**B**–**D**) Imino proton spectrum of *htel27[Br22]* (**B**) after 5 min in D_2O, (**C**) after 2 h in D_2O, and (**D**) after 5 h in D_2O.

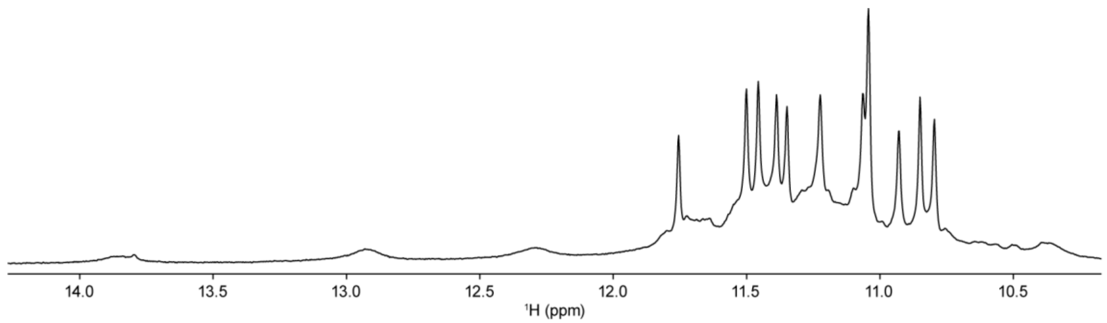

Figure S8. 1D NMR imino proton spectra of *htel27[Br22]* in ~100 mM Na^+ at 25 °C.

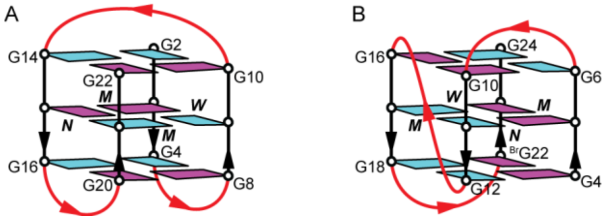

Figure S9. Schematic structures of human telomeric G-quadruplexes in Na^+ solution: (**A**) *htel22* and (**B**) *htel27[Br22]*.
